# Genome Analysis of a Novel Tembusu Virus in Taiwan

**DOI:** 10.3390/v12050567

**Published:** 2020-05-22

**Authors:** Shih-Huan Peng, Chien-Ling Su, Mei-Chun Chang, Huai-Chin Hu, Su-Lin Yang, Pei-Yun Shu

**Affiliations:** Center for Diagnostics and Vaccine Development, Centers for Disease Control, Ministry of Health and Welfare, Taipei City 11561, Taiwan; shpeng@cdc.gov.tw (S.-H.P.); sue@cdc.gov.tw (C.-L.S.); newlover@cdc.gov.tw (M.-C.C.); huaichinhu@cdc.gov.tw (H.-C.H.); cerline@cdc.gov.tw (S.-L.Y.)

**Keywords:** Tembusu virus, Sitiawan virus, flavivirus 3′-UTR variable region

## Abstract

We identified and isolated a novel Tembusu virus (TMUV) strain TP1906 (TMUV-TP1906) from a *Culex* *annulus* mosquito pool collected from the northern part of Taiwan in 2019. The TMUV-TP1906 genome is a 10,990-nucleotide-long, positive-sense, single-stranded RNA, consisting of a single open reading frame (ORF) encoding a polyprotein of 3425 amino acids, with 5′ and 3′ untranslated regions (UTRs) of 94 and 618 nucleotides, respectively. The nucleotide sequence of the TMUV-TP1906 of ORF exhibited 93.71% and 91.27% similarity with Sitiawan virus (STWV) and the TMUV prototype strain MM1775, respectively. The 3′-UTR variable region of TMUV-TP1906 showed nucleotide sequence divergence with other TMUV strains. Phylogenetic analysis of the complete ORF and polyprotein sequences revealed that TMUV-TP1906 is most closely related to STWV which causes encephalitis and retarded growth in chickens. We found that the TMUV-TP1906 caused a cytopathic effect (CPE) in the DF-1 chicken fibroblast cell line, while no apparent CPE was observed in Vero and C6/36 cells. In this study, we first identified and isolated a novel TMUV strain in Taiwan. In addition, to our knowledge, it is the first time that the TMUV strain was isolated from the *Cx. annulus* mosquitoes. Further study is warranted to investigate the host range and virulence of TMUV-TP1906.

## 1. Introduction

Tembusu virus (TMUV) is a single-stranded, positive-sense RNA virus that belongs to the *Flaviviridae* family, Flavivirus genus and Ntaya virus group. The TMUV prototype strain MM1775 (TMUV-MM1775) was first isolated from *Culex tritaeniorhynchus* mosquitoes in Kuala Lumpur, Malaysia in 1955. Since 2000, many TMUV strains (TMUVs) have been isolated from birds and mosquitoes, including chicks, ducks, geese, pigeons, sparrows and *Culex* mosquitoes. TMUV consists of several genetically closely related virus strains with noticeable pathogenicity for poultry, such as Sitiawan virus (STWV), duck TMUV (DTMUV), Perak virus and Baiyangdian virus [1]. STWV was isolated from sick broiler chicks in Sitiawan District of Perak State, Malaysia in 2000 [2]. STWV-infected chicks showed encephalitis, growth retardation and increased blood sugar levels. In 2010, DTMUV, also called duck egg-drop syndrome virus, was found to extensively infect ducks with high morbidity (up to 90%) and mortality (5% to 30%) rates in southeast China [3,4]. DTMUV outbreaks also occurred in layer and broiler duck farms in Pekin ducks in Malaysia in 2012 and in Thailand in 2013 [5,6].

The TMUV genome is approximately 11,000 nucleotides and contains 5′ and 3′ untranslated regions (UTRs), and a long open reading frame (ORF) that encodes three structural proteins (capsid (C), pre-membrane protein (prM) and envelope (E)) and seven nonstructural (NS) proteins (NS1, NS2A, NS2B, NS3, NS4A, NS4B, and NS5). Phylogenetic analysis of the nucleotide sequences of the ORFs of several TMUVs showed that TMUV-MM1775 and STWV clustered together away from the genomes of other DTMUVs [7].

TMUVs have been isolated from several mosquito species in Asia, mainly *Cx. tritaeniorhynchus* [8,9] and also *Culex* species, including *Cx. vishnui*, *Cx. gelidus* and *Cx. pipiens* [5,9,10]. A mosquito vector competence study by O’Guinn et al. demonstrated that *Cx. vishnui* developed high viral titers after feeding on TMUV-infected chicks and could readily transmit TMUV to naive chickens [11]. Although TMUV are considered mosquito-borne viruses [4], in vivo studies suggested that TMUVs could be transmitted among ducks by direct contact and aerosol transmission [12,13]. The role of mosquitoes as vectors in the transmission of TMUV to avian hosts still needs further study.

Taiwan is an island off the southeastern coast of mainland China that is near the epicenter of DTMUV outbreaks. Taiwan straddles the Tropic of Cancer, having a warm tropical-subtropical climate and large variety of mosquito species and is also an important rest point for migrating birds [14]. To monitor arboviruses in Taiwan, we conducted a survey on mosquitoes collected from wetlands, pig farms and parks in Taiwan between April and September 2019. In this study, we identified novel TMUV strains in *Cx. annulus* and *Cx. tritaeniorhynchus* mosquitoes collected from northern and central parts of Taiwan, respectively. We isolated and characterized the genetic sequence of this novel virus.

## 2. Materials and Methods

### 2.1. Collections of Mosquitoes

**Figure 1:**
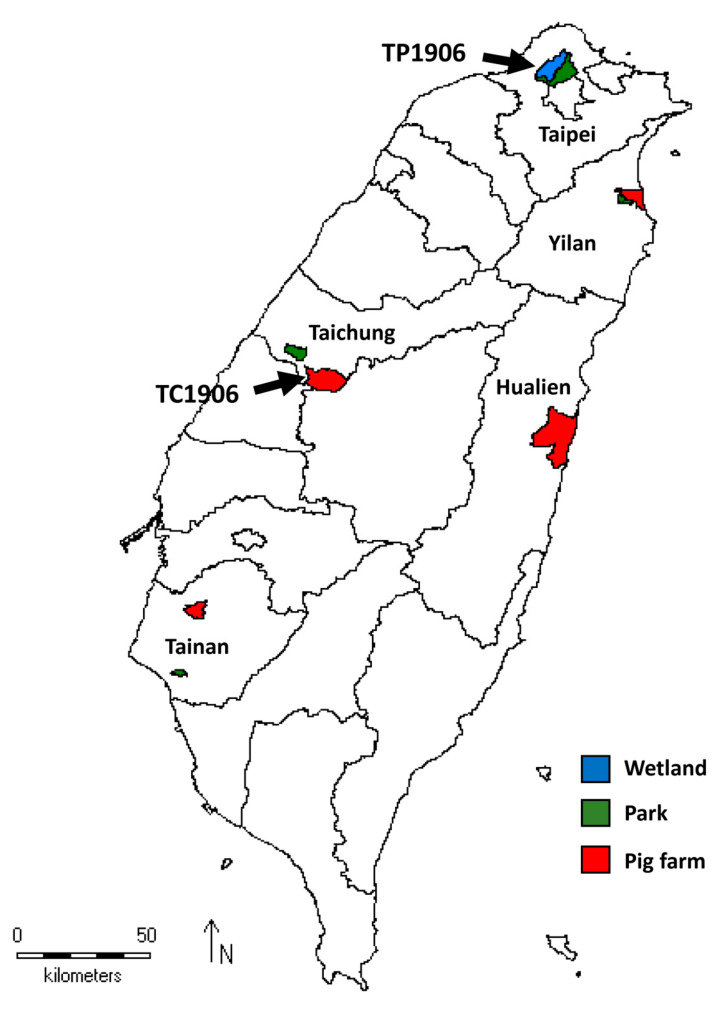
Map showing mosquito collection sites in Taiwan. Mosquitoes collected from wetland, parks and pig farms are indicated with blue, green and red colors, respectively. Black arrows indicate collection sites of mosquitoes infected with novel TMUV strains TP1906 and TC1906.

The mosquitoes were collected on pig farms near rice paddy fields in the northern (Wujie Township in Yilan County), central (Wufeng District in Taichung City), southern (Xiaying District in Tainan City), and eastern (Shoufeng Townships in Hualien County) regions; from a wetland habitat for waterbirds in the northern (Beitou District in Taipei City) region; and also from community parks in the northern (Shilin District in Taipei City and Luodong Township in Yilan County), central (Nantun District in Taichung City) and southern (North District in Tainan City) regions of Taiwan between April and September, 2019 (Figure 1). The mosquitoes were collected using dry ice traps in wetland and parks and sweep nets in pig farm and were transported either alive or on dry ice to the laboratory. Mosquito collections by sweep nets were conducted only on the same day between 18:30 and 20:30 in the pig farms, while dry ice traps were set up overnight from 17:00 to 8:00 the next morning in wetland and parks as previously described [15]. Only female mosquitoes were analyzed in this study. The mosquitoes were frozen at –20 °C and further examined and classified according to the characteristics of the species. The pooled (1 to 50) mosquitoes were used for RNA extraction and virus isolation. The mosquito pools were homogenized in a TissueLyzer (Qiagen GmbH, Hilden, Germany) with two cycles at 4 °C for 90 sec at a frequency of 30 Hz after adding a 3 mm steel ball to each tube and 0.6 mL RPMI 1640 liquid medium. The pools were then clarified by centrifugation. The supernatants were sterilized by filtration and removed for RNA extraction and virus isolation.

### 2.2. RNA Extraction and Real Time RT-PCR

Viral RNA was extracted from the mosquito suspensions or cell culture supernatants using the QIAamp viral RNA mini kit (Qiagen GmbH, Hilden, Germany). The flavivirus-specific primers (1370F: 5′-TGY GTB TAC AAC ATG ATG GG; 1442F: 5′-ATA TGG TAC ATG TGG CTA GGA GC and 1620R: 5′-GTG TCC CAN CCH GCT GTG TCA) were mixed and used for the RT-PCR screening assay. Real-time RT-PCR was performed using a QuantiTect SYBR Green RT-PCR kit (Qiagen GmbH, Hilden, Germany) with the following parameters: 50 °C for 30 min; 95 °C for 15 min and 45 cycles of 95 °C for 15 s, 55 °C for 30 s, 72 °C for 20 s, and 77 °C for 20 s; and 95 °C for 1 min, melting curve program from 68 °C to 95 °C [16,17]. DNA sequencing of positive RT-PCR products was performed, and RT-PCR positive mosquito pools were subjected to virus isolation.

### 2.3. Virus Isolation and Immunofluorescence Assay (IFA)

Filtered homogenates of mosquito pools were initially cultured with the *Aedes albopictus* C6/36 cell line or Vero cell line. C6/36 cells were infected with TMUV-TP1906 and tested by IFA. Briefly, cells were fixed and incubated with anti-flavivirus monoclonal antibody (D56.3) for 1 h. The cells were subsequently washed and incubated with FITC-conjugated goat anti-mouse IgG antibody (Invitrogen, Carlsbab, CA, USA) for 1 h. After washing, the images of the cells were examined using a fluorescence microscope (Carl Zeiss, Oberkochen, Germany).

### 2.4. Viral Growth Kinetics

C6/36, Vero, BHK-21 and DF-1 cell lines were obtained from the American Type Culture Collection (ATCC; www.atcc.org) and used for the viral growth kinetics assays. C6/36 cells were grown in RPMI1640 medium supplemented with 5% fetal bovine serum (FBS) (Gibco, Invitrogen, Carlsbab, CA, USA), 100 units/mL–0.1 mg/mL–0.25 μg/mL penicillin-streptomycin-amphotericin B (PSA) (Gibco, Invitrogen), 1X non-essential amino acids (NEAA) (Gibco, Invitrogen) and incubated in 5% CO_2_ at 30 °C. Vero cells were grown in M199 medium supplemented with 5% FBS-PSA-NEAA and incubated in 5% CO_2_ at 37 °C. BHK-21 cells were cultured in MEM medium supplemented with 4% FBS-PSA-NEAA and incubated in 5% CO_2_ at 37 °C. DF-1 cells were cultured in DMEM medium supplemented with 10% FBS-PSA in 5% CO_2_ at 39 °C. Growth curves were performed by inoculating 0.1 mL TMUV-TP1906 (3.12 × 10^8^ viral copies) into 80% confluent cells (5 × 10^5^ cells per well) in 6-well plates (Corning, NY, USA). After 1 h incubation, medium with virus was removed and added 3 mL of fresh medium. All cell culture supernatants were collected at 0, 6, 24, 48, 72, 96 and 120 h post infection and the cell cultures were observed daily for cytopathic effect under a light microscopy (Carl Zeiss, Oberkochen, Germany). The viral RNA extracted from the supernatants was quantified by real time RT-PCR with the flavivirus-specific primer set and the viral copy number calculated according to the standard curve. All experiments were performed in triplicate.

### 2.5. Complete Genome Sequencing

RNA extracted from the cell culture supernatants and pellets from the first cell culture passage were used for RT-PCR and DNA sequencing. RT-PCR was performed using the Superscript III One-Step RT-PCR system with Platinum Taq High Fidelity (Invitrogen, Carlsbab, CA, USA). The RT-PCR parameters were as follows: 55 °C for 30 min; 94 °C for 2 min and 40 cycles of 94 °C for 15 s, 50 °C or 54 °C for 30 s, and 68 °C for 1 min; and a prolonged final extension at 68 °C for 5 min. The cDNA of the 5′ end of the viral genome was generated by rapid amplification of cDNA ends (RACE) using a 5′/3′ RACE kit, 2nd generation (Roche, Mannheim, Germany) with a gene specific primer (GSP-5′ end, Table 1). For the 3′ end, polyadenylated (polyA) tails were added to the 3′ ends of virus genomic ssRNA using T4 RNA ligase. Tailing reactions were performed at 37 °C for 1 h and used for RT-PCR directly. The 5′ and 3′ end fragments were amplified using the genome-specific primer GSP-5′ end and 3′UTR-10 with oligo d(T)-anchor primer (provided by the 5′/3′ RACE kit), respectively (listed in Table 1). The PCR products were sequenced directly by using the BigDye Terminator Cycle Sequencing Kit and the ABI 3730xl DNA analyzer (Applied Biosystems) according to the manufacturer’s protocols. Each forward and reverse primer was used for sequencing, and the overlapping sequences were combined. The sequence identity of the ORF and protein sequence were analyzed by BLAST (https://blast.ncbi.nlm.nih.gov/Blast.cgi). The sequence identities of the 3′-UTR and 3′-UTR variable region were analyzed by Clustal Omega (https://www.ebi.ac.uk/Tools/msa/clustalo/).

### 2.6. Phylogenetic Analysis

Genome sequences of TMUVs were aligned, edited, and analyzed using Clustal W software [20]. The complete open reading frame sequences of TMUVs including sequences representing the most closely related strains to the TMUV strain TP1906 isolated in this study were obtained using BLAST and retrieved from GenBank for phylogenetic analysis (Table 2). The phylogenetic analysis was performed using MEGA version 7 (http://www.megasoftware.net/) [21]. To construct the phylogenetic trees, the maximum likelihood method using the Tamura–Nei model (Figures 3 and 5) and the maximum likelihood method using the JTT matrix-based model (Figure 4) were utilized. The reliability of the analysis was evaluated by a bootstrap test with 1000 replications. Sequences of Bagaza virus (GenBank numbers: KR108245 and MF380425), Japanese encephalitis virus (GenBank number: KF667310) and West Nile virus (GenBank number: M12294) were used as the outgroup.

## 3. Results

### 3.1. Identification of Novel TMUV Strains from Culex Mosquitoes in Taiwan

Mosquitoes were collected from nine localities in Taiwan from April to September 2019 (Figure 1). A total of 15,374 mosquitoes were collected and analyzed. Table 3 shows a summary of the mosquito species, numbers and RT-PCR results. Nineteen mosquito species from nine genera of the *Culicidae* family were identified. The most commonly collected species was *Cx. tritaeniorhychus* (70.90%, *n* = 10,900), followed by *Cx. annulus* (11.87%, *n* = 1825) and *Mansonia uniformis* (5.45%, *n* = 838). A flavivirus-specific primer set targeting the NS5 gene (amplicon is approximately 200 bp) was initially used for screening the mosquito pools by real-time RT-PCR. A total of 429 mosquito pools were tested, and 55 pools were positive; RT-PCR products were then sequenced and analyzed using the NCBI BLAST platform. Of the 55 positive pools, 53 pools contained genomic Japanese encephalitis virus (JEV) sequences, while a *Cx. annulus* mosquito pool (TP1906) collected from a wetland habitat for waterbirds in the Beitou District, Taipei City, in northern Taiwan, and a *Cx. tritaeniorhynchus* mosquito pool (TC1906) collected from a pig farm near rice paddy fields in Wufeng Township, Taichung City in central Taiwan, contained TMUV-like sequences. The partial NS5 gene sequences of RT-PCR products detected in the TP1906 and TC1906 mosquito pools were 100% identical and are closely related to STWV (GenBank number: JX477686) and TMUV-MM1775 (GenBank number: MH414569) sequences with 92.9% and 92.31% nucleotide similarities, respectively.

To further study whether the novel flaviviruses detected in *Cx. annulus* and *Cx. tritaeniorhychus* mosquito pools were identical, we amplified a partial NS1 gene sequence (964 bp) by using a TMUV NS1-specific primer set (TMUV-NS1_F and TMUV-NS1_R, in Table 1) [19]. The amplified NS1 gene sequences from these two mosquito pools were 99.79% identical with two nucleotide differences. The sequence obtained from the *Cx. tritaeniorhynchus* mosquito pool (TC1906) had a serine at amino acid position 131 of the NS1 protein (GenBank number: MN958524), while the *Cx. annulus* mosquito pool (TP1906) had an asparagine at position 131 (GenBank number: MN747003). The results indicated that the viruses from these two mosquito pools were very similar.

### 3.2. Isolation of a Novel TMUV from the Cx. Annulus Mosquito Pool

The C6/36 and Vero cell lines were used for propagation of the novel TMUV from the TP1906 and TC1906 mosquito pools. The novel TMUV strain TP1906 was successfully isolated from the *Cx. annulus* mosquito pool (TP1906). Virus isolation from the *Cx. tritaeniorhynchus* mosquito pool (TC1906) was unsuccessful.

### 3.3. Different Cell Lines Were Permissive for TMUV-TP1906 Infection

**Figure 2:**
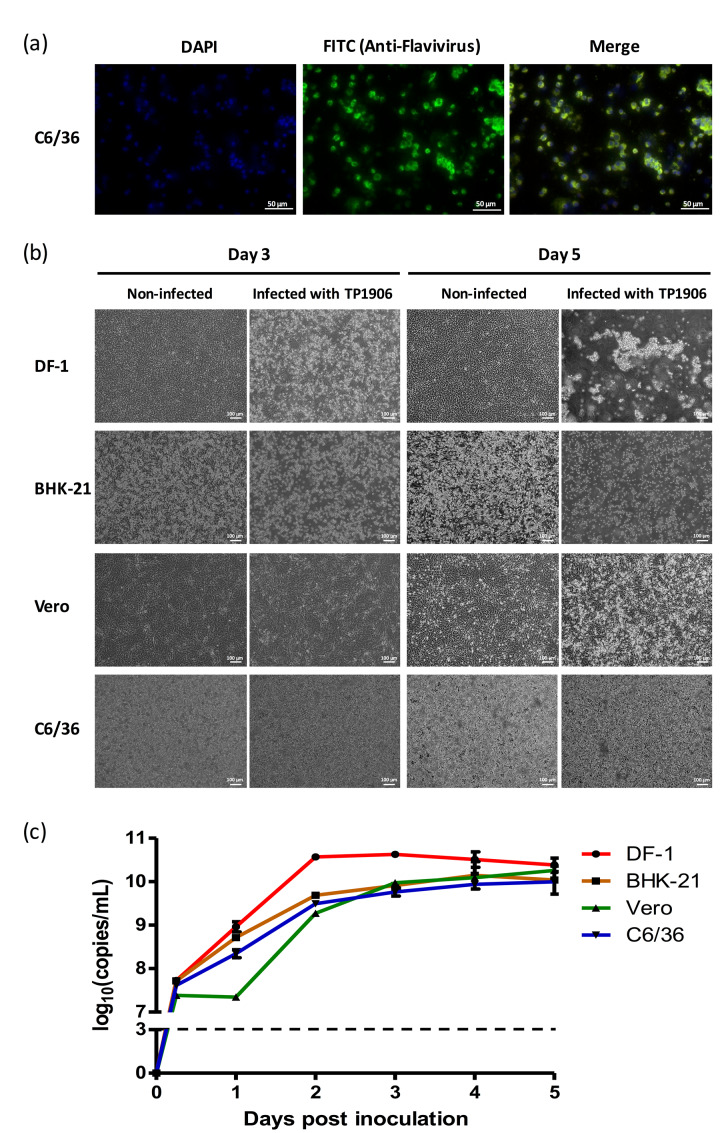
Different cell lines were permissive to TMUV-TP1906 infection. (**a**) The TMUV-TP1906 infection was detected by IFA in C6/36 cells. (**b**) The morphology of TMUV-TP1906 infected cells at 3 days and 5 days postinfection. (**c**) Growth curves of TMUV-TP1906 in different cell lines. All the experiments were performed in triplicate. Dashed line indicates the limit of detection of real time RT-PCR by using the flavivirus-specific primer set.

Immunofluorescence assay using anti-flavivirus antibody indicated that the TMUV-TP1906 was capable of infecting C6/36 cells (Figure 2a). To determine whether the TMUV-TP1906 replication can take place in different cell lines derived from mosquito (C6/36), monkey (Vero), hamster (BHK-21) and chicken (DF-1), we inoculated the TMUV-TP1906 to these cell lines and observed the cytopathic effects and detected viral RNA by using real-time RT-PCR. Although lacking a noticeable cytopathic effect (CPE) in C6/36 and Vero cell cultures, we observed apparent CPE in DF-1 and BHK-21 cell lines 72 h postinfection (Figure 2b). Notably, all the infected DF-1 and BHK-21 cells were detached five days postinfection. The TMUV-TP1906 was capable of replication in DF-1 cells and reached its highest titer at 48 h postinfection (Figure 2c). In contrast, the virus replication was relatively slowly in Vero cells compared to the BHK-21 and C6/36 cells.

### 3.4. Characteristics of the Genome Sequence of TMUV Strain TP1906

The 10990-nucleotide-long whole genome sequence of TMUV-TP1906 (GenBank number: MN747003) was determined to contain a 5′- UTR (94 nt), capsid(C; 360 nt), pre-membrane protein (prM; 501 nt), envelope (E; 1503 nt), nonstructural protein 1 (NS1; 1056 nt), NS2A (681 nt), NS2B (393 nt), NS3 (1857 nt), NS4A (378 nt), 2K (69 nt), NS4B (762 nt), NS5 (2718 nt); and 3′-UTR (618 nt). The sequence length of the ORF of TMUV-TP1906 was similar to that of other TMUVs, but the lengths of the 5′-UTR and 3′-UTR varied in different TMUVs. Table 4 shows comparative analyses of multiple alignments of different gene regions between TMUV-TP1906 and other TMUVs, including the STWV, TMUV-MM1775, DTMUV-SD14 and DTMUV strains. The results indicated that the ORF sequence of TMUV-TP1906 had 93.71%, 91.27% and 88% similarities to those of the STWV, TMUV-MM1775 and DTMUV-SD14 strains, respectively. The 5′-UTR (94 nt) sequence of TMUV-TP1906 had 94.68% and 92.55% similarities to those of the TMUV-MM1775 and DTMUV-SD14 strains, respectively. The 3′-UTR (618 nt) sequence was more conserved among TMUVs and had 95.15% and 92.72% similarity to the TMUV-MM1775 and DTMUV-SD14 strains, respectively.

### 3.5. Phylogenetic Analysis of TMUV-TP1906 and Other Flaviviruses Based on ORF Sequences

**Figure 3:**
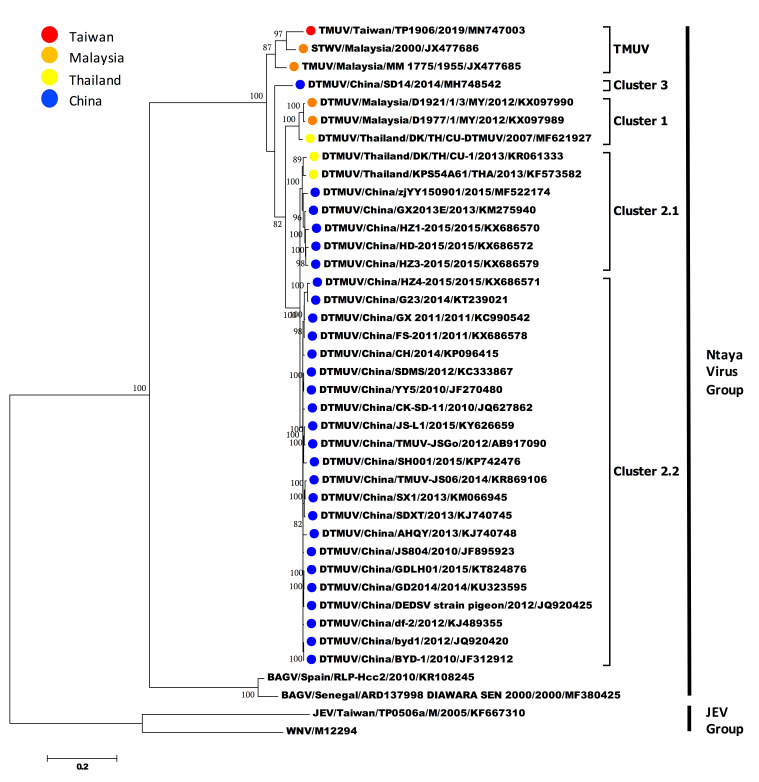
Phylogeny of TMUV-TP1906, Tembusu virus strains (TMUVs) and duck Tembusu virus strains (DTMUVs) based on the ORF (10,278 nt). The evolutionary history was inferred by using the maximum likelihood method based on the Tamura–Nei model [22]. The tree with the highest log likelihood is shown. The percentage of trees in which the associated taxa clustered together is shown next to the branches. Initial tree(s) for the heuristic search were obtained automatically by applying Neighbor-Join and BioNJ algorithms to a matrix of pairwise distances estimated using the maximum composite likelihood (MCL) approach, and then selecting the topology with superior log likelihood value. The tree is drawn to scale, with branch lengths measured in the number of substitutions per site. The analysis involved 40 nucleotide sequences. All positions containing gaps and missing data were eliminated. Evolutionary analyses were conducted in MEGA7 [21]. The reliability of the analysis was calculated using 1000 bootstrap replication. Bootstrap support values >75 are shown. The scale bar indicates nucleotide substitutions per site.

To understand the phylogenetic relationship between TMUV-TP1906 and other mosquito-borne flaviviruses, the complete ORF sequences of 40 representative flaviviruses, namely 36 TMUVs (listed in Table 2), Bagaza virus (GenBank numbers: KR108245 and MF380425), Japanese encephalitis virus (GenBank number: KF667310) and West Nile virus (GenBank number: M12294), were analyzed. Phylogenetic analysis indicated that TMUVs could be divided into two lineages (lineages TMUV and DTMUV). The TMUV lineage contained TMUV-MM1775, STWV, and TMUV-TP1906, and TMUV-TP1906 was most closely related to STWV. The DTMUV lineage could be further grouped into three clusters (clusters 1 to 3) (Figure 3). Cluster 1 contained DTMUV strains from Malaysia and Thailand. Clusters 2.1 and 2.2 contained many avian DTMUV strains found in Thailand and China. DTMUV-SD14 isolated from mallards (*Anas platyrhynchos*) in China was classified into cluster 3.

### 3.6. Amino Acid Sequence Comparison between TMUV-TP1906 and Other TMUVs

**Figure 4:**
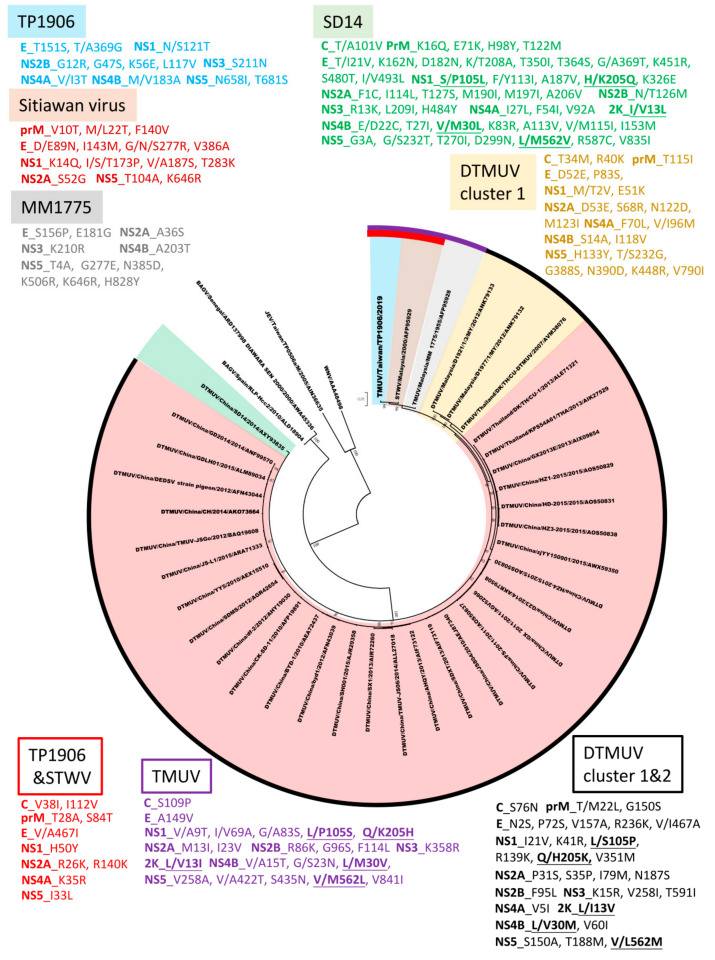
Phylogeny of TMUV-TP1906, Tembusu virus strains (TMUVs), and duck Tembusu virus strains (DTMUVs) based on the polyprotein amino acid sequences (3425 aa). The evolutionary history was inferred by using the maximum likelihood method based on the JTT matrix-based model [23]. The tree with the highest log likelihood is shown. Initial tree(s) for the heuristic search were obtained automatically by applying Neighbor-Join and BioNJ algorithms to a matrix of pairwise distances estimated using a JTT model, and then selecting the topology with superior log likelihood value. The tree is drawn to scale, with branch lengths measured in the number of substitutions per site. The analysis involved 40 amino acid sequences. All positions containing gaps and missing data were eliminated. Evolutionary analyses were conducted in MEGA7 [21]. The reliability of the analysis was calculated using 1000 bootstrap replication. Bootstrap support values >75 are shown. The scale bar indicates amino acid substitutions per site. The unique amino acid residues in TP1906, Sitiawan virus, MM1775, SD14, and DTMUV cluster 1 compared to other TMUV strains were colored in blue, brown, gray, green, and yellow, respectively. The unique amino acid residues in DTMUV cluster 1 and 2, TMUV lineage, and TP1906 and STWV compared to other TMUV strains were colored in black, purple, and red, respectively.

Genomic sequences of TMUV-TP1906 and other TMUV strains contain a single ORF that encodes a polypeptide of 3425 amino acids, except the ORFs of two DTMUV strains (D1977 and D1921) that encode 3424 amino acids with a deletion in the NS5 protein at amino acid position 86. The ORFs of TMUV-TP1906 and other TMUVs shared 86.52%–93.71% similarity at the nucleotide level and 95.71%–98.92% similarity when the amino acid sequences of the polyproteins were compared (Table 4). The amino acid sequences of the prM, E, and NS1 proteins of TMUV-TP1906 showed the highest similarity with TMUV-MM1775, while sequences of the C, NS2A, NS3, NS4A, NS4B, and NS5 proteins of TMUV-TP1906 showed the highest similarity with STWV. Figure 4 shows the phylogenetic relationship based on the amino acid sequences of polyproteins of TMUV-TP1906 and TMUVs. The results indicated that TMUV-TP1906 is most closely related to STWV.

### 3.7. Sequence Alignment and Phylogenetic Analysis of 3′-UTR Variable Region of TMUV-TP1906 and Other TMUVs

**Figure 5:**
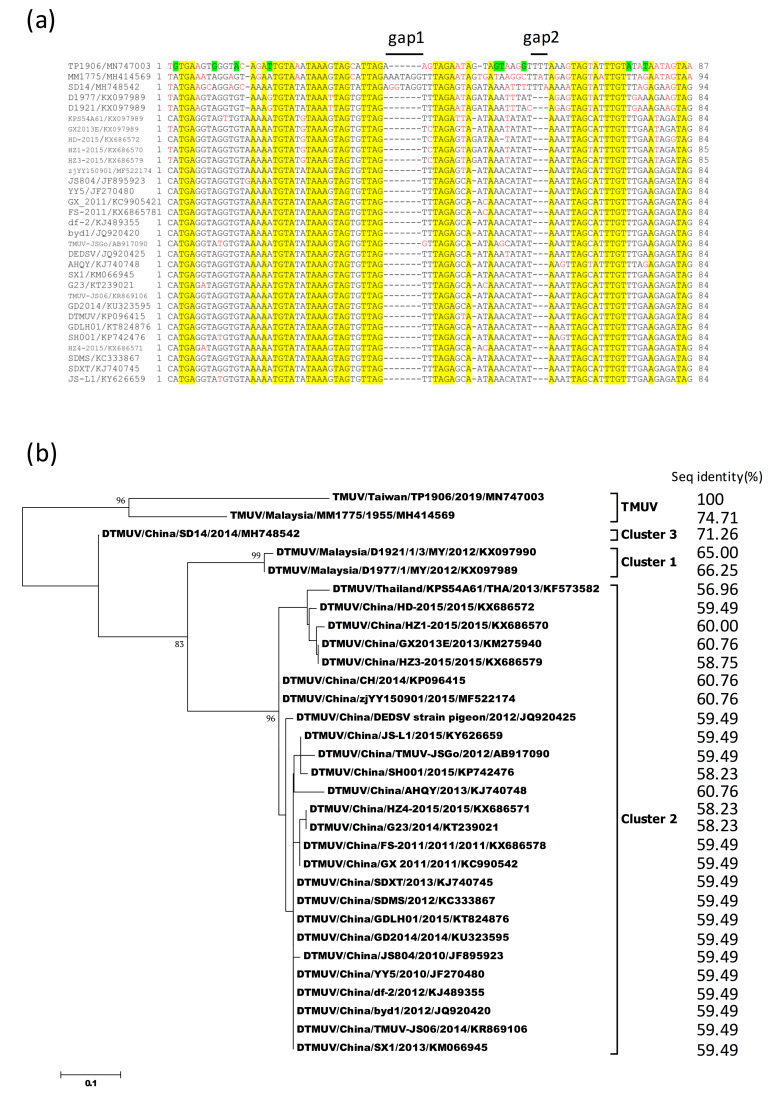
Sequence alignment and phylogenetic tree of 3′UTR variable regions of TMUV-TP1906, Tembusu virus strains (TMUVs), and duck Tembusu virus strains (DTMUVs). (**a**) 3′UTR sequence alignment of TMUV-TP1906, TMUVs, and DTMUVs. The conserved nucleotides are highlighted in yellow and the unique nucleotides of TMUV-TP1906 are highlighted in green. The nucleotides different from the consensus sequence are colored in red; (**b**) phylogeny of TMUV-TP1906, TMUVs and DTMUVs based on 3′UTR variable region. The evolutionary history was inferred by using the maximum likelihood method based on the Tamura–Nei model. The reliability of the analysis was calculated using 1000 bootstrap replication. Bootstrap support values >75 are shown. The scale bar indicates amino acid substitutions per site. Sitiawan virus and four DTMUV strains including DK/TH/CU-1, BYD-1, CK-SD-11, and DK/TH/CU-DTMUV which do not deposit the 3′-UTR sequences in GenBank were not analyzed.

Comparison of 5′-UTR and 3′-UTR sequences between TMUV-TP1906 and other TMUVs showed 90.43%–94.68% and 89.28%–95.15% sequence similarities, respectively (Table 4). Analysis of 3′-UTR variable region (VR) sequences (lengths varied from 84 to 94 nucleotides) between TMUV-TP1906 and other TMUVs showed that TMUV-TP1906 contained 1 gap (gap 1) and DTMUVs contained 2 gaps (gaps 1 and 2), while no gaps were found in the VR sequences of TMUV-MM1775 and DTMUV-SD14 (Figure 5a). The novel TMUV-TP1906 had a 3 nucleotide insertion in gap 2 and a nucleotide insertion in gap 1. In addition, there were nine unique nucleotide substitutions in the VR of TMUV-TP1906 compared with those of the other TMUVs. The 3′-UTR VR sequences were more consistent among cluster 2 of the DTMUV lineage, but more variable in the TMUV lineage and clusters 1 and 3 of the DTMUV lineage (Figure 5b). Comparisons of the 3′-UTR VR sequence of TMUV-TP1906 with those of TMUV-MM1775, DTMUV-SD14 (cluster 3), DTMUV-D1921 (cluster 1), and DTMUV-D1977 (cluster 1) showed 74.71%, 71.26%, 65.00%, and 66.25% sequence similarities, respectively, and 56.96%–60.76% sequence similarity with cluster 2 of DTMUVs. Figure 5b shows the phylogenetic tree based on the 3′-UTR VR sequences of TMUVs, and the overall topology was similar to the phylogenetic tree based on analysis of ORF sequences (Figure 3).

## 4. Discussion

In this study, we identified two novel TMUV strains, TMUV-TP1906 and TMUV-TC1906, from *Cx. annulus* and *Cx. tritaeniorhynchus* mosquitoes collected from a wetland in northern Taiwan and a pig farm in central Taiwan, respectively. TMUV-TP1906 was isolated from the *Cx. annulus* mosquito pool and grew well in Vero and C6/36 cells without significant cytopathic effects. Through virus isolation and comprehensive genomic and protein sequence analysis, we found that this novel flavivirus is a TMUV-related virus and is most closely related to STWV. STWV, DTMUV, and Bagaza virus, which belong to Ntaya serocomplex virus; and JEV and West Nile virus, which belong to JEV serocomplex virus, have been shown to be primarily *Culex* mosquito-associated viruses that cause severe diseases in avian species [7]. Whether TMUV-TP1906 is a pathogen in birds or other animals needs further study.

Phylogenetic analysis based on the complete ORF of TMUVs showed that TMUVs can be divided into two lineages, TMUV and DTMUV. Analysis of amino acid sequences showed that there are 22 amino acid substitutions unique to the TMUV lineage and 29 amino acid substitutions unique to cluster 1 and cluster 2 of the DTMUV lineage (Figure 4). The DTMUV-SD14 strain which belongs to cluster 3 of the DTMUV lineage, could be considered as a transitional group between the TMUV lineage and clusters 1 and 2 of the DTMUV lineage. The DTMUV-SD14 strain contains 48 amino acid mutations, which are different from other TMUVs. In addition, there are five informative amino acid substitutions (NS1-105, NS1-205, 2K-13, NS4B-30 and NS5-562) for the differentiation of the TMUV lineage, DTMUV-SD14 (cluster 3 of the DTMUV lineage) and clusters 1 and 2 of the DTMUV lineage. The TMUV lineage contains three virus strains, the TMUV-MM1775 prototype strain, STWV, and TMUV-TP1906. TMUV-MM1775 has not been documented as a pathogen in birds or other animals; however, STWV, which is most closely related to TMUV-TP1906, causes encephalitis and retards the growth of chicks. Analysis of amino acid sequences showed that TMUV-TP1906 contains 12 unique amino acid substitutions compared with other TMUVs. In addition, comparison of protein sequences of TMUV-TP1906 and STWV with other TMUVs showed 10 amino acid substitutions. These informative amino acid positions may be useful for the classification of TMUVs and may play crucial roles in the evolution and protein function of TMUVs.

The Asn glycosylation site at residue 154 of the E protein (154-Asn) of TMUVs is critical for virus tissue tropism and transmissibility in poultry [24]. TMUV-MM1775 contains 156-Pro, which can disrupt the N-linked glycosylation of 154-Asn of the E protein, and thus may affect viral virulence and transmissibility in avian species [24]. Except for TMUV-MM1775, all the other TMUVs, including TMUV-TP1906, contain 156-Ser which would not disrupt 154-Asn glycosylation and thus may not affect the glycosylation-associated infectivity of TMUVs in avian species. A recent study showed that the Thr to Lys mutation at residue 367 of the E protein plays a predominant role in viral cell-adaptation and virulence attenuation in ducks [25]. TMUV-TP1906 contains 367-Thr of the E protein and thus may retain the virulence in the host. In addition to E protein, NS1 protein contains three N-linked glycosylation sites at residues 130, 175, and 207. A recent study showed that some DTMUV strains had a glycosylation motif mutation at residues 175–178 from NTTD to NITD but the glycosylation site at 130 was conserved [19]. Interestingly, we found NS1 glycosylation motif at residues 175 and 207 were conserved in TMUV-TP1906 and TMUV-TC1906, however, TMUV-TC1906 had a glycosylation motif mutation at residues 130–133 from NNTF to NSTF. Whether these mutations affect protein functions or virulence needs further study.

The 5′ and 3′ UTRs of flaviviruses are important for virus replication and transmission [26]. The 5′ end of the 3′UTR of flavivirus is known to have a VR with different nucleotide lengths [26], and this VR sequence may be associated with the production of subgenomic flavivirus RNA and immune modulation, hence contributing to virus adaptation in vector and non-vector hosts [27,28]. The 3′UTR VRs of TMUVs consist of 84 to 94 nucleotides immediately downstream of the ORF. Phylogenetic analysis of 3′UTR VR and ORF sequences showed similar topological features, indicating that 3′UTR VRs may serve as a potential marker for TMUV evolution. Sequence analysis also showed that the 3′UTR VR of TMUV-TP1906 is very unique among TMUVs (Figure 5). More research is needed to understand the genetic diversity and function of the 3′UTR VR of TMUVs.

Prominent CPE was observed in BHK-21, C6/36, Vero and DF-1 cell lines after infection with DTMUV [5,10,18,29]. STWV infection induced CPE in the BK3 cells (chicken bursal lymphoma cell line) but not in CPK (porcine kidney cell line), MARC-145 (monkey kidney cell line) and Vero cells [2]. In our study, we found that the TMUV-TP1906, which is closely related to STWV, grew in C6/36 and Vero cell without inducing significant CPE, however, the virus caused drastic CPE in DF-1 and BHK-21 cell lines (Figure 2).

TMUVs are emerging pathogenic flaviviruses causing severe avian diseases in Malaysia, Thailand, and China. Taiwan is located south of East Asia and is close to the epicenter of DTMUV outbreaks in China. In this study, we first identified a TMUV in Taiwan. Interestingly, TMUV-TP1906 is most closely related to STWV and TMUV-MM1775 found in Malaysia and is different from the DTMUVs found in China (Figure 3 and Figure 4 Figure 3; Figure 4). Previous study has divided TMUVs into the Chinese mainland TMUV lineage and Southeast Asian TMUV lineage [8]. According to the phylogeny, TMUV-MM1775, STWV, TMUV-TP1906, and two TMUV strains identified from *Cx. tritaeniorhynchus* in Yunnan Province of China were grouped as the Southeast Asian TMUV lineage. The phylogeographical analysis suggested that the TMUVs might spread from Southeast Asian countries such as Malaysia and Thailand to the Yunnan Province of China (southern China) and further spread to areas in northern China such as Shandong Province. However, the mechanism of long-distance spread of TMUVs is still unknown. Liu et al. suggested that the spread of TMUVs might occur via migratory birds and ornithophilic *Culex* spp. mosquitoes, since TMUVs have been identified in wild birds such as pigeons and mallards (*Anas platyrhynchos*) [7]. The East Asian–Australasian flyway is one of the world’s great flyways for migratory birds, and the flyway passes through many countries including Malaysia, Thailand, China, and Taiwan; thus, it is possible that the spread of TMUVs might be through this flyway. In addition, it has been suggested that the spread of avian influenza virus and JEV might also occur through this flyway [30,31].

In this study, we reported the first isolation of a novel TMUV strain from *Culex* mosquitoes in Taiwan. In addition, it is the first time that the TMUV strain was isolated from *Cx. annulus* mosquitoes. We performed comprehensive genomic and amino acid analyses between TMUV-TP1906 and other TMUVs, and our results may help to increase understanding of the diversity and evolution of TMUVs. Further study is warranted to investigate the virulence and epidemiology of TMUV-TP1906 in Taiwan.

## Figures and Tables

**Table 1:** Primers used in RT-PCR and DNA sequencing for TMUV-TP1906.

Name	Sequence (5′ to 3′)	Amplicon (bp)	Annealing Temp (°C)	References
P1f	AGAAGTTCRYCTGTGTGA	638	50	[6,18]
DF_R638	CAGCAGTCTATGTCTTCAGG			
DF_F441	CGATAGTTGCTGGGCTGAAGC	675	50	[6]
DF_R1115	GCAGTAAGATCTCACAACCGC			
DF_F954	GCTTCAGCTGTCTGGGGATGC	696	50	
DF_R1650	CAATGACTCTTTGTTTTGCCACG			
DF_F2353	GGCACTGCTATTGTGGATGGG	987	50	
DF_R3339	GGTGGGGTGGTGCAAGACC			
DF_F3302	GGAACAACTGTCACAGTAACG	1393	50	
DF_R4694	GCATGACTCCCACTCCAGCC			
DF_F4406	GCATCACAGAGATTTGATGTGG	757	54	
DF_R5162	CCTGAACCTGGATGTAGGTCC			
DF_F4874	GCAAGTCATCGTCGTGCAACC	709	50	
DF_R5582	GCTCTTCAATGTCTGTTATTGGC			
DF_F5399	GCTCACACCTCAGCGAGTGC	851	50	
DF_R6249	GGTCATTGTAACTTATCCCAGC			
DF_F6494	CGCTCACAGAATGACAGAATCC	855	50	
DF_R7348	GGAACATCTGTAGCCACTATGC			
DF_F6807	GAACCAGAGAGACAGAGATCGC	1352	50	
DF_R8158	CCCTAGCTAGCCATTCCTCGG			
DF_F7940	GCAGGTTCAGGAAGTGAGAGG	597	50	
DF_R8536	GGATTGTCTTGGTCATAATGCC			
DF_F8383	GGATGCACAAAACCAACCGC	833	50	
DF_R9215	GGCCGAGATGTCACGCAGC			
DF_F9274	GGGACACTAGAATAACCAAGGC	1212	50	
DF_R10485	CCAACATCCGGTGGCAGGG			
TMUV-E_F	TTCAGCTGTCTGGGGATGCA	1503	50	[19]
TMUV-E_R	GGCATTGACATTTACTGCCA			
TMUV-NS1_F	GACACGGGGTGCTCAATCGACTT	1056	50	
TMUV-NS1_R	AGCCATGACCTTTGATTTGAT			
TMUV-NS3_F	GGAGGAGTCATCTGGGATGTG	1857	50	
TMUV-NS3_R	TCTCTTTCCACTCGCAAAATC			
TMUV-NS5_F	GAACTGGCAGAACTTTGGGGGAG	2711	50	
TMUV-NS5_R	TTACAAGACACCTTCACTCCAGC			
1_F	AAATGACTTCAGGACACCTC	723	50	This study *
1_R	ACATACCTTGTCCACACTTC			
2_F	ATGTCATGGATCACTCAAGG	400	50	
2_R	CAGTCAAGTCAATGCTGTTG			
3_F	AAAAGAAAGGAGGCATGCTA	523	50	
3_R	GGAACATCCCATATGACTCC			
5_F	CAGTCGGAAGTGCATTAAAC	683	54	
5_R	CAGCTGTAGTCAGCATGTAT			
7_F	TTAGAATCCTGTCAAAGCCC	767	50	
7_R	CACCTTCACCACCTTATTCA			
8_F	CTGGAATCTCGTTGATAGGG	572	50	
8_R	TAATGAGTTGAACGCACAGA			
3′UTR-2_F	CCAACCCTCAATAGGTTCAA	612	50	This study ^†^
3′UTR-2_R	GAGGGTCTCCTAGTCTATCC			
3′UTR-5_F	ATACATGGAAGACAAGACCC	465	50	
3′UTR-5_R	GAGACGGTATTGAACGCTTA			
3′UTR-10_F	AGGAGCTAAGCGTTCAATAC	427	50	
3′UTR-10_R	GACTCTGTGTTCTACCACC			
GSP-5′ end	GGTCGCCTCACTGACCCCAACTAGC	331	55	For RACE
3′UTR-10_F	AGGAGCTAAGCGTTCAATAC	424	55	
oligo d(T)-anchor	GACCACGCGTATCGATGTCGACT(16)V		55	

* Design based on Sitiawan virus cDNA sequence (JX477686); **^†^** design based on M1775 virus cDNA sequence (MH414569).

**Table 2:** Tembusu virus strains used for phylogenetic analysis in this study.

TMUV /DTMUV Strains	Host	Location	Year/ Month	GenBank Accession Number /ORF Sequence Identity (%)/ Length (Nucleotides)	GenBank Accession Number /Protein Sequence Identity (%)/ Length (Amino Acids)
TP1906	*Cx. annulus*	Taipei/ Taiwan	2019/ Jun	MN747003 100 (10,990)	100 (3425)
TC1906 ^†^	*Cx. tritaeniorhynchus*	Taichung/ Taiwan	2019/ Jun	MN958524 NS1:99.79 (964)	NS1:99.69 (964)
Sitiawan virus	Broiler Chicks	Perak state/ Malaysia	2000	JX477686 93.71 (10,278)	AFP95929 98.92 (3425)
MM1775	*Cx. tritaeniorhynchus*	Kuala Lumpar/ Malaysia	1955	JX477685 91.27 (10,278)	AFP95928 98.57 (3425)
YY5	Duck	Zhejiang Province/ China	2010	JF270480 87.05 (10,990)	AEX15510 96.50 (3425)
BYD-1	Duck	China	2010	JF312912 87.15 (10,278)	AEA72437 96.55 (3425)
JS804	Goose	Jiangsu Province/ China	2010	JF895923 87.12 (10,990)	AEJ87340 96.38 (3425)
CK-SD-11	Chicken	China	2010	JQ627862 87.01 (10,278)	AFP19891 96.41 (3425)
GX_2011	Duck	Guangxi Province/ China	2011	KC990542 87.05 (10,990)	AGV52066 96.47 (3425)
FS-2011	Duck	China	2011	KX686578 87.00 (10,991)	AOS50837 96.44 (3425)
df-2	Duck	China	2012	KJ489355 87.16 (10,990)	AHY19030 96.53 (3425)
byd1	Duck	Hebei Province/ China	2012	JQ920420 87.15 (10,990)	AFN43039 96.55 (3425)
JSGo	Goose	China	2012	AB917090 87.21 (10,959)	BAQ19608 96.53 (3425)
DEDSV strain pigeon	Pigeon	Beijing Autonomous City/ China	2012	JQ920425 87.09 (10,990)	AFN43044 96.61 (3425)
AHQY	Layer Duck	China	2013	KJ740748 86.65 (10,990)	AIF73122 96.35 (3425)
SX1	Chicken	China	2013	KM066945 86.80 (10,990)	AIR72260 95.97 (3425)
G23	Goose	China	2014	KT239021 86.92 (10,881)	AKR79508 96.61 (3425)
JS06	Chicken	China	2014	KR869106 86.69 (10,990)	ALL27018 95.7 (3425)
GD2014	Duck	China	2014	KU323595 87.06 (10,990)	ANF99570 96.50 (3425)
DTMUV/CH/2014	Duck	China	2014	KP096415 87.05 (10,990)	AKO73664 96.44 (3425)
GDLH01	Duck	China	2015	KT824876 87.07 (10,990)	ALM89034 96.47 (3425)
SH001	Duck	Shanghai Province /China	2015	KP742476 87.10 (10,990)	AJR29358 96.50 (3425)
HZ4-2015	Broiler Duck	China	2015	KX686571 86.63 (10,991)	AOS50830 95.94 (3425)
SD14	*Anas platyrhynchos*	China	2014	MH748542 88.00 (11,001)	AXY93835 96.64 (3425)
DK/TH/CU-DTMUV	Duck	Thailand	2007	MF621927 87.21 (10,278)	AVM38076 96.41 (3425)
D1977/1/MY	Pekin Duck	Malaysia	2012	KX097989 87.09 (10,988)	ANK79132 96.20 (3424)
D1921/1/3/MY	Pekin Duck	Malaysia	2012	KX097990 87.10 (10,988)	ANK79133 96.18 (3424)
DK/TH/CU-1	Duck	Thailand	2013	KR061333 86.91 (10,278)	ALE71321 96.55 (3425)
KPS54A61	Duck	Thailand	2013	KF573582 86.84 (10,990)	AIK27529 96.35 (3425)
GX2013E	Duck	China	2013	KM275940 86.97 (10,990)	AIX09854 96.23 (3425)
HD-2015	Layer Duck	China	2015	KX686572 86.75 (10,991)	AOS50831 96.15 (3425)
HZ1-2015	Layer Duck	China	2015	KX686570 86.52 (10,990)	AOS50829 96.12 (3425)
HZ3-2015	Duck	China	2015	KX686579 86.94 (10,992)	AOS50838 96.18 (3425)
zjYY150901	Duck	China	2015	MF522174 86.82 (10,990)	AWX59350 96.29 (3425)
SDMS	*Culex* Mosquito	Shangdong Province/China	2012	KC333867 87.04 (10,990)	AGR42654 96.47 (3425)
SDXT	Layer Duck	China	2013	KJ740745 87.08 (10,990)	AIF73119 96.58 (3425)
JS-L1	Duck	China	2015	KY626659 87.14 (10,890)	ARA71333 96.53 (3425)

**^†^** The sequence identity of TC1906 partial NS5 (156 nt) and NS1 (964 nt) was 100% and 99.79% with TP1906, respectively.

**Table 3:** Summary of the mosquito species, number of mosquitoes, and pools tested and positive Tembusu virus pools.

Species	Taipei		Taichung		Tainan		Yilan		Hualien	Num Individuals	Num Pools	Num TMUV Pos Pools
	Park	Wetland	Park	Pig Farm	Park	Pig Farm	Park	Pig Farm	Pig Farm			
*Aedes aegypti*	0	0	0	0	7	0	0	0	0	7	3	0
*Aedes albopictus*	86	110	4	0	33	0	43	0	33	309	23	0
*Aedes malikuli*	4	0	0	0	0	0	0	0	0	4	3	0
*Aedes penghuensis*	0	9	0	0	0	0	0	0	0	9	4	0
*Aedes vexans*	0	75	0	0	0	0	0	0	0	75	5	0
*Anopheles sinensis*	0	0	0	106	0	3	0	12	35	156	8	0
*Anopheles tessellatus*	0	179	2	0	0	0	0	0	10	191	7	0
*Armigeres subalbatus*	5	40	0	0	105	0	0	0	66	216	14	0
*Coquillettidia crassipes*	0	2	0	0	0	0	0	0	0	2	1	0
*Culex annulus*	4	606	22	7	52	2	645	15	472	1825	50	1 *
*Culex fuscocephala*	0	0	0	0	0	2	0	0	12	14	3	0
*Culex pipiens form molestus*	63	19	53	0	87	0	17	0	21	260	21	0
*Culex quinquefasciatus*	15	11	29	0	174	7	116	0	133	485	25	0
*Culex sitiens*	0	0	0	0	62	0	0	0	4	66	5	0
*Culex tritaeniorhynchus*	2	2470	71	1301	914	2280	327	1473	2062	10,900	231	1*
*Heizmannia taiwanensis*	4	0	0	0	0	0	0	0	0	4	2	0
*Mansonia uniformis*	0	809	0	0	0	0	0	0	29	838	19	0
*Tripteroides bambusa*	0	11	0	0	0	0	0	0	0	11	4	0
*Uranotaenia novobscura*	0	2	0	0	0	0	0	0	0	2	1	0
Total	183	4343	181	1414	1434	2294	1148	1500	2877	15,374	429	2

* 50 *Culex annulus* and 46 *Culex tritaeniorhynchus* were in the two TMUV positive pools, respectively.

**Table 4:** Comparison of genomic sequences between TMUV-TP1906, Sitiawan virus and other Tembusu virus strains.

Genomic Region (% Sequence Identity)	TMUV-TP1906	Sitiawan Virus (JX477686)	TMUV-MM1775 (MH414569)	DTMUV-SD14 (MH748542)	DTMUVs (see Table 2)
Complete genome sequence	10,990 nt (100)	NA ^#^	11,001 (91.54)	11,001 (88.31)	10,890–10,992 * (86.93–87.49)
5′-UTR	94 nt (100)	NA ^#^	94 nt (94.68)	94 nt (92.55)	93–95 (90.43–93.68)
3′-UTR (total length)	618 nt (100)	NA ^#^	629 nt (95.15)	629 nt (92.72)	618 nt (89.28–92.14)
3′-UTR (variable region)	87 nt(100)	NA ^#^	94 nt (74.71)	94 nt (71.26)	84–85 nt (56.96–66.25)
ORF	10,278 nt (100)	10,278 nt (93.71)	10,278 nt (91.27)	10,278 nt (88.00)	10,275–10,278 nt ^†^ (86.52–87.21)
polyprotein	3425 aa (100)	3425 aa (98.92)	3425 aa (98.57)	3425 aa (96.64)	3424–3425 aa (95.71–96.61)
C	360 nt (100) 120 aa (100)	360 nt (96.67) 120 aa (99.17)	360 nt (94.71) 120 aa (96.67)	360 nt (90.53) 120 aa (95.00)	360 nt (88.89–90.28) 120 aa (93.33–95.00)
prM	501 nt (100) 167 aa (100)	501 nt (91.20) 167 aa (98.20)	501 nt (90.80) 167 aa (98.80)	501 nt (87.00) 167 aa (95.21)	501 nt (84.40–85.40) 167 aa (94.61–96.41)
E	1503 nt (100) 501 aa (100)	1503 nt (93.68) 501 aa( 98.40)	1503 nt (90.21) 501 aa (98.60)	1503 nt (87.62) 501 aa (96.41)	1503 nt (86.09–86.96) 501 aa (96.41–97.8)
NS1	1056 nt (100) 352 aa (100)	1056 nt (93.93) 352 aa (97.73)	1056 nt (91.86) 352 aa (98.86)	1056 nt (89.67) 352 aa (96.31)	1056 nt (85.13–88.35) 352 aa (90.62–94.89)
NS2A	681 nt (100) 227 aa (100)	681 nt (92.22) 227 aa (99.12)	681 nt (89.88) 227 aa (97.8)	681 nt (86.43) 227 aa (93.81)	681 nt (84.14–85.61) 227 aa (90.75–92.51)
NS2B	393 nt (100) 131 aa (100)	393 nt (91.09) 131 aa (96.95)	393 nt (89.57) 131 aa (96.95)	393 nt (87.28) 131 aa (93.13)	393 nt (85.24–86.77) 131 aa (90.84–93.89)
NS3	1857 nt (100) 619 aa (100)	1857 nt (94.18) 619 aa (99.52)	1857 nt (91.76) 619 aa (99.03)	1857 nt (89.01) 619 aa (98.71)	1857 nt (87.51–88.26) 619 aa (97.58–98.71)
NS4A	378 nt (100) 126 aa (100)	378 nt (93.39) 126 aa (99.21)	378 nt (90.74) 126 aa (98.41)	378 nt (86.21) 126 aa (96.03)	378 nt (84.62–87.04) 126 aa (92.86–96.03)
2K	69 nt (100) 23 aa (100)	69 nt (95.65) 23 aa (100)	69 nt (94.12) 23 aa (100)	69 nt (94.12) 23 aa (95.65)	69 nt (85.51–91.18) 23 aa (95.65)
NS4B	762 nt (100) 254 aa (100)	762 nt (93.83) 254 aa (99.61)	762 nt (90.94) 254 aa (99.21)	762 nt (85.9) 254 aa (95.28)	762 nt (84.83–88.52) 254 aa (91.34–97.64)
NS5	2715 nt (100) 905 aa (100)	2715 nt (94.18) 905 aa (99.34)	2715 nt (91.71) 905 aa (98.56)	2715 nt (88.32) 905 aa (97.79)	2712–2715 nt ^†^ (87.18–87.81) 904–905 aa ^†^ (96.91–98.34)

# NA: Not available. Sequence is not available in GenBank database; * 5 DTMUV strains including DK/TH/CU-1, BYD-1, CK-SD-11, DK/TH/CU-DTMUV and G23 which do not deposit complete genome sequences in GenBank were not analyzed; † NS5 protein of both DTMUV D1977 and D1921 strains composed of 904 amino acids
